# Assessing Retinal Structure in Patients with Parkinson’s Disease

**DOI:** 10.4172/2155-9562.1000485

**Published:** 2019-03-07

**Authors:** Jonathon B Young, Pooja Godara, Vesper Williams, Phyllis Summerfelt, Thomas B Connor, Sergey Tarima, Alexis Visotcky, Robert F Cooper, Karen Blindauer, Joseph Carroll

**Affiliations:** 1 Department of Ophthalmology and Visual Sciences, Medical College of Wisconsin, Milwaukee, USA; 2 Division of Biostatistics, Institute for Health and Equity, Medical College of Wisconsin, USA; 3 Department of Biomedical Engineering, Marquette University, Milwaukee, USA; 4 Department of Neurology, Medical College of Wisconsin, USA; 5 Department of Cell Biology, Neurobiology and Anatomy, Medical College of Wisconsin, USA

**Keywords:** Optical coherence tomography, Parkinson’s disease, Fovea, Retina

## Abstract

**Objective:**

The retina is an extension of the central nervous system (CNS), and ocular symptoms can precede manifestations of CNS disorders. Given that several neurodegenerative conditions that affect the brain exhibit ocular symptoms, the retina may be an accessible biomarker to monitor disease progression. Dopamine, the key neurotransmitter related to Parkinson’s disease (PD), is contained in amacrine and interplexiform cells, which reside in specific retinal layers. Understanding how loss of dopaminergic cells affects retinal anatomy could be relevant for monitoring disease progression. Here, our objective is to evaluate retinal structure (foveal pit morphology and thickness) in patients with PD.

**Methods:**

Thirty-three Caucasian subjects diagnosed with PD and 40 age-matched Caucasian control subjects underwent retinal imaging with spectral-domain optical coherence tomography (SD-OCT). Axial length measurements were used to correct the lateral scale of each macular volume scan. From these corrected volumes, foveal morphology was quantified with previously described algorithms, and Early Treatment Diabetic Retinopathy Study (ETDRS) grids of retinal thickness were generated and incorporated into a logistic regression model to predict PD.

**Results:**

Interocular foveal morphology measurements were highly symmetrical in PD patients and control subjects. There were no significant differences in foveal pit morphology between PD patients and control subjects. Using a model incorporating sex and axial length corrected ETDRS regions, we generated a receiver operating characteristic curve with a C-statistic of 0.80.

**Conclusion:**

Our study, which to our knowledge is the first to properly scale OCT measurements when quantifying retinal thickness, demonstrates that PD patients retain foveal symmetry between eyes. When constructing a model to predict PD, sex, along with the center 1 mm and temporal outer ETDRS regions, were significant predictors of PD. In addition to proper scaling of OCT measures, gender and racial differences in retinal anatomy should be considered in building future predictive PD models when using OCT.

## Introduction

Parkinson’s disease (PD) is a progressive neurological disorder resulting from selective dopaminergic neuronal loss in the substantia nigra, first described by James Parkinson in 1817 [1]. Parkinson’s disease is the second most common neurodegenerative disorder following Alzheimer’s Disease [2], and the prevalence of diagnosis is expected to double by 2040 [3]. Given the developmental origin of the retina, it is not surprising that there are a variety of visual symptoms associated with PD, including abnormal contrast sensitivity, motion perception abnormalities, impaired visual acuity, color vision deficits, and visual hallucinations [4,5]. The loss of retinal dopaminergic cells is thought to underlie the visual dysfunction observed in PD [6]. Dopamine is contained in amacrine and interplexiform cells, which reside in the inner nuclear layer (INL) and inner plexiform layer (IPL) of the retina, respectively [7]. Understanding how loss of dopaminergic cells affects retinal anatomy in PD could be relevant for monitoring disease progression, assessing therapeutic response to treatment, or facilitating early detection of the disease.

The human retina can be examined non-invasively using spectral domain optical coherence tomography (SD-OCT) [8,9], thus this technique has been proposed as a way to monitor PD within the retina [10–29]. There are a large number of studies using SD-OCT in PD patients, but they have provided conflicting results. For example, retinal thickness has been suggested by some to be reduced in PD patients versus controls [10,15,18], while others report no significant difference in total retinal thickness [11,21,24]. A major advantage of SD-OCT is its ability to resolve individual cellular layers in the retina, though even sublayer analyses in PD patients have yielded discrepant results. Many studies have documented retinal nerve fiber layer (RNFL) thinning in PD patients versus age-matched controls [15–18, 22,23,25,30], though other reported no significant RNFL thinning[10,19,21]. Results from other inner retinal layers are similarly variable. For example, thinning of the inner retinal layers including the ganglion cell layer (GCL) and IPL of PD patients has been reported [13,24]. Lee et al. observed thinning of the INL [21], while Garcia-Martin et al. observed increased thickness in the INL with thinning in the GCL and IPL when comparing PD patients with healthy subjects [15]. Živković et al. and Sari et al. both documented statistically significant thinning in the GCL-IPL layer compared to controls [26,27]. Sari et al. noted an inverse correlation with PD severity and duration, while Živković et al. did not observe this occurrence. In a five year retinal reevaluation using SD-OCT, Satue et al. found significant changes compared to baseline in 7 of 9 Early Treatment Diabetic Retinopathy Study (ETDRS) thickness regions, and 2 of 6 regions of the RNFL [28].

Foveal morphology, which represents in some way the topography of multiple retinal layers, has also been studied in patients with PD. Spund et al. provided data showing that the foveal pit becomes broader and thinner in patients with PD [12] and Ding et al. showed that PD patients could be discriminated from normal controls based on foveal morphology, with a specificity of 70% [14].

Three factors might contribute to the discrepant findings in previous studies. First, previous reports did not take into account inter-individual variability in axial length. Variability in axial length can affect the lateral scale of OCT data from some devices and scaling each measurement to the patient’s axial length is crucial to ensure the accuracy of each measurement. For example, Odell et al. showed that failing to correct for axial length in data from the Cirrus HD-OCT can lead to errors in the ETDRS thickness measurements of greater than 40 microns [31]. To the best of our knowledge, no commercially available OCTs correct for inter-individual differences in axial length when generating macular thickness maps. If the retina is going to be used as a biomarker for PD, the sensitivity of this measurement needs to be as accurate as possible to prevent misclassification of a condition. Second, the type of OCT device used has not been consistent in all forms of retinal evaluation with PD. Statistically significant differences in specific macular regions and correlations with disease duration detected on one device may not be statistically significant when using an alternate SD-OCT machine [17,18]. Third, differences in retinal layer segmentation (automated vs. manual) could contribute to discrepant results.

The purpose of the present study was to use axial length scaled SD-OCT volumes to compare foveal morphology and retinal thickness in PD patients versus age-matched control subjects. These retinal evaluations were then placed into a logistic regression model and cross-validation analysis was performed to determine our accuracy in predicting PD. Based on these findings, we propose that OCT devices enable either an export of raw thickness data for offline manipulation or an onboard correction of individual differences in ocular biometry. From these data files, algorithms can be applied to better predict the diagnosis of PD, potentially offering earlier detection of this condition.

## Methods

### Subjects

All research involving human subjects followed the tenets of the Declaration of Helsinki and was approved by the Institutional Review Board at the Medical College of Wisconsin. PD patients were referred by a neurologist and asked to participate in our study following his or her neurology exam. All subjects gave informed consent after explanation of risks and possible benefits of the study. A total of 62 patients diagnosed with PD were recruited for this study, along with 40 age-matched healthy control (HC) subjects. PD and HC subjects were excluded from the study if previous retinal pathology had been documented by a physician or was identified via a self-report questionnaire. Twenty-nine of the 62 PD patients were excluded from further analyses for a variety of reasons including: patient consented but then refused ocular imaging, poor OCT signal quality, artifacts in OCT macular thickness map, and ocular pathology detected (epiretinal membrane, glaucoma) or reported via a self-report questionnaire. Thirty-three Caucasian patients diagnosed with PD and 40 age-matched Caucasian control subjects were further analyzed for this work.

Both eyes were imaged in PD patients. Of the 40 HC subjects, 7 had only one eye included in the study due to available data or low signal quality. OCT scans were also reviewed for retinal abnormalities, and the left eye of 1 PD subject with a retinal abnormality was excluded from the analysis. The left eye of 3 additional PD subjects was excluded from analysis due to concurrent self-reported ocular pathology. The average age of PD patients (±1SD) was 64.6 ± 10.1 years. The average age of HC subject was 61.9 years ± 10.9 years. Table 1 provides additional demographic data for PD patients. PD patients self-reported ocular history, but age of PD diagnosis and current medications were confirmed by completing a subject chart review. The motor Unified Parkinson’s Disease Rating (UPDR) Scale scores were documented in 28 of 33 PD subjects to indicate disease severity (Table 1). The average age at PD diagnosis was 60.5 years. If the age of the PD diagnosis could not be confirmed, the age of the subject’s first visit to Froedtert & the Medical College of Wisconsin was reported. The average time from PD diagnosis until time of imaging was 4.1 years.

Color vision was tested with the Richmond HRR test [32] and/or Neitz Test of Color Vision [33]. For the PD patients, 14/33 made one or more errors on the Neitz test. All who made errors were subsequently tested using the Richmond HRR test, on which they made no errors. Color vision testing for normal controls was not uniform, as they were included from various previous studies, with two subjects not having any documented color vision testing performed. Of the remaining 38 normal controls, two made errors on both the Neitz and Richmond HRR tests (one making errors consistent with a protan defect and the other making mild nonspecific red/green errors). In addition, 14 normal controls made one or more errors on the Neitz test. Four of these 14 were subsequently tested with the HRR and made no errors.

### SD-OCT imaging & measuring foveal pit morphology

Volumetric images of the macula were obtained using the CirrusTM HD-OCT (Carl Zeiss Meditec, Dublin, California, USA) system. Exclusion criteria included diagnosis of retinal pathology, OCT signal quality below 7/10, and inability to maintain stable fixation as documented by segmentation errors found in OCT scans. For PD patients, macular scan quality averaged 9.27 ± 0.90 for the right eye, and 9.48 ± 0.62 for the left eye. In controls, macular scan quality averaged 9.28 ± 0.81 for the right eye and 9.45 ± 0.86 for the left eye. SD-OCT scans were reviewed by an ophthalmologist for any signs of retinal pathology, and seven of the 33 PD patients showed class one drusen [34]. As described in Wagner-Schuman et al., we corrected the lateral scale of all OCT data sets for inter-individual differences in axial length [35]. We obtained axial length measurements using low coherence interferometry (IOLMaster, Carl Zeiss Meditec). We multiplied 6mm (nominal scan length) by the ratio of the subject’s axial length to that assumed by the CirrusTM HD-OCT system (24.46 mm) in order to derive the actual scan lengths. For PD patients, axial length measurements ranged from 21.40 to 27.89 mm. Consequently, actual scan lengths measured 5.25 to 6.84 mm. For HC subjects, axial length measurements ranged from 21.55 to 26.95 mm, and therefore actual scan lengths measured from 5.29 to 6.61 mm.

As previously described [35], the location of the fovea within each volume scan was identified automatically using the onboard fovea-finder algorithm of the Cirrus. The coordinates of the foveal center and the retinal thickness data from the volume scans were exported for offline analysis (Cirrus Research Browser; Carl Zeiss Meditec). Custom software was created to generate revised Early Treatment Diabetic Retinopathy Study (ETDRS) thickness maps (MatLab; The MathWorks, Natick; MA), which incorporated the actual axial length and retinal thickness data for each subject. Each thickness map used for analysis was aligned to the center of the fovea, not necessarily the center of the OCT volume. Foveal pit morphology was quantified using a previously described difference-of-Gaussian algorithm [36].

### Statistical Analysis

Statistical analysis was performed using GraphPad Prism (GraphPad, La Jolla, CA) and R 3.3.1 (www.r-project.org). The D’Agostino & Pearson normality test was used to assess if these measurements were normally distributed. Since all foveal metrics were normally distributed, Bland-Altman plots [37–38] and a Student’s t-test were used to analyze the data. Values of p<0.05 would indicate statistically significant findings.

To build a parsimonious model predicting PD, the forward variable selection method was used to identify a model with the smallest Akaike information criteria (AIC). Then, the predictive properties of this model were evaluated with the C statistic (Area under Receiver Operating Characteristic {ROC} Curve). The ROC curve along with the C-statistic were internally validated using leave one out cross-validation technique (LOOCV). Due to previous studies showing that females have thinner retinas, sex was later added as an additional predictor to this model in subsequent analysis, which led to a new parsimonious model. Similarly, LOOCV cross-validation was applied to the ROC and the C-statistic.

## Results

### Foveal pit morphology remains symmetrical in PD

**Figure 1:**
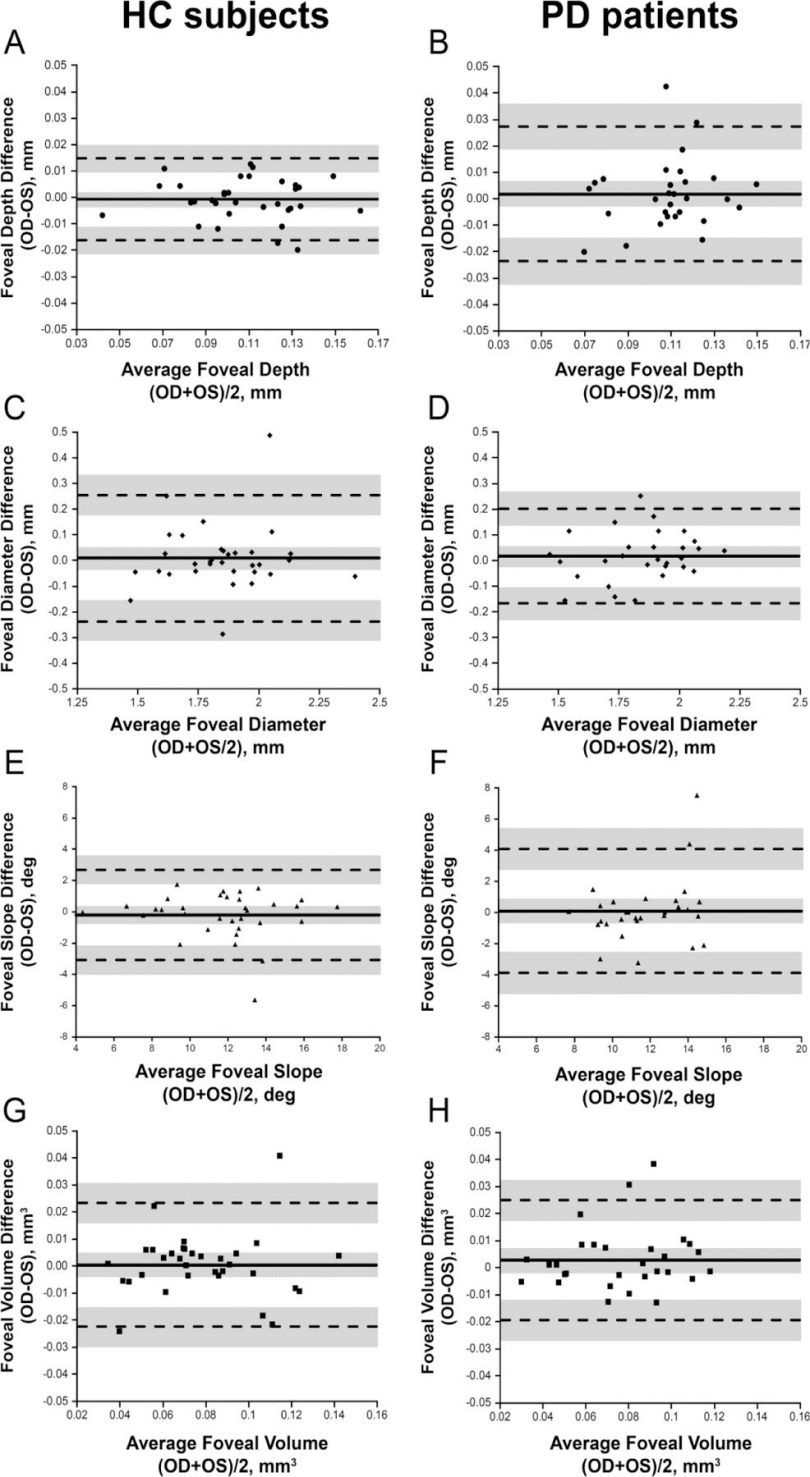
Interocular symmetry of foveal morphology in HC Subjects (A, C, E, G)) and PD Patients (B, D, F, H). Bland-Altman plots for foveal pit depth (A, B), diameter (C, D), slope (E, F), and volume (G, H). For PD patients, the absolute mean difference between eyes (OD minus OS; solid line in each plot) was less than 0.0019 mm for pit depth, less than 0.0171 mm for pit diameter, less than 0.095 degrees for foveal slope, and less than 0.0029 mm^3^ for foveal volume. For HC subjects, the absolute mean difference was less than 0.0007 mm for pit depth, less than 0.0095 mm for pit diameter, less than 0.2063 degrees for foveal slope, and less than .0006 mm3 for foveal volume. The mean difference is represented by the solid black line, while the dashed lines represent the 95% limits of agreement (LOA) for the bias. Shaded regions represent the confidence limits on the bias and LOA. There was no apparent relationship between the mean difference and the magnitude of the measurement.

We first compared foveal pit morphology between PD patients and HC subjects and determined if measurements were symmetrical between eyes. To test this, we used a Bland-Altman plot for all foveal metrics calculations to determine the agreement between eyes. As shown in Figure 1, the mean difference between eyes was close to zero for all foveal pit parameters. Only one HC subject and one PD patient fell outside the limits of agreement, and the similarity between measurements appears to indicate a lack of foveal rearrangement between eyes.

Following confirmation of interocular symmetry between eyes of PD patients and HC subjects, the foveal metrics were subsequently compared between groups (Table 2). There was no statistical difference in any of the foveal metrics assessed. Together, these results suggest the fovea retains interocular symmetry in our sample of PD patients.

### Logistic regression models to predict Parkinson’s disease

**Figure 2:**
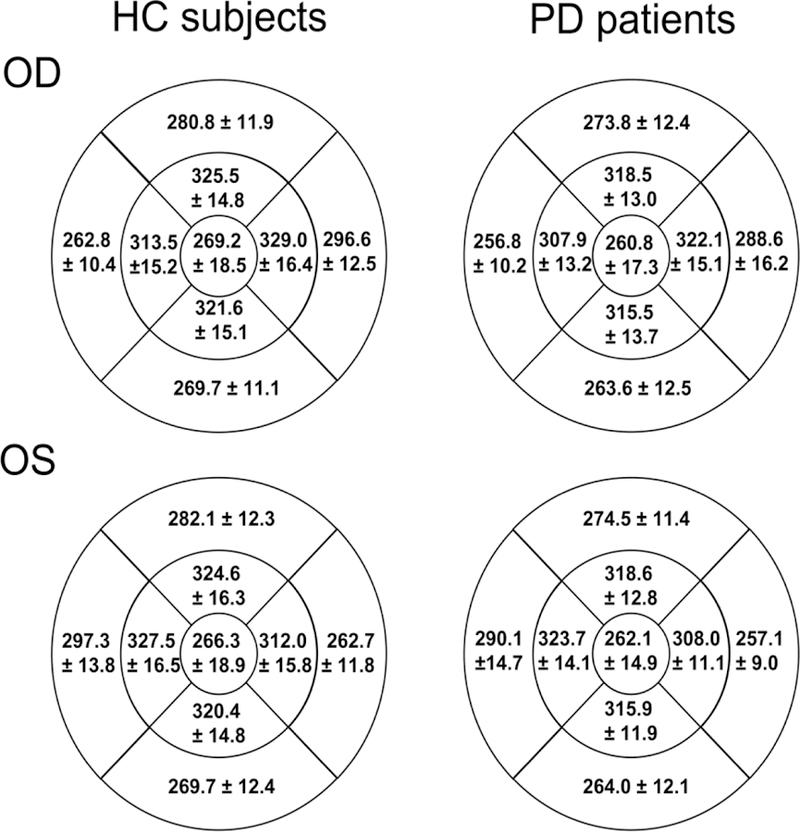
Ocular biometry corrected Early Treatment Diabetic Retinopathy Study (ETDRS) thickness maps for HC subjects and PD patients. PD patients have thinner retinas compared to HC subjects. The ETDRS measurements of all eyes were included in the analysis to help establish collinear predictors of PD. The measurements of all ETDRS regions were highly correlative as shown in Supplementary Table 2.

As the fovea remains symmetrical in our observed cases of PD, we continued to investigate other methods to use the retina as a biomarker for PD investigations. The axial length corrected ETDRS regions of HC subjects and PD patients are shown in Figure 2. Using the ETDRS regions, we investigated the correlation of all measurements for all eyes included in this study, shown in Supplementary Table 1. Due to the high correlation among all measurements, no regions were further collapsed into a single case. We then incorporated logistic regression models using the forward selection method, AIC, and C-statistic criteria for modeling of PD. First, a model containing the 9 measurements in Supplementary Table 1 were generated, and only the superior outer ETDRS region remained in the model as a predictor of PD. Next, the effects of foveal metrics (depth, diameter, and slope) were modeled, and none were significant predictors of PD. Lastly, all 9 measures including foveal metrics were considered for model entry, and again the only significant predictor of PD was the superior outer ETDRS region measurement with the C-statistic at 0.662. The cross-validated C-statistic did not change, and after rounding was also 0.662.

**Figure 3:**
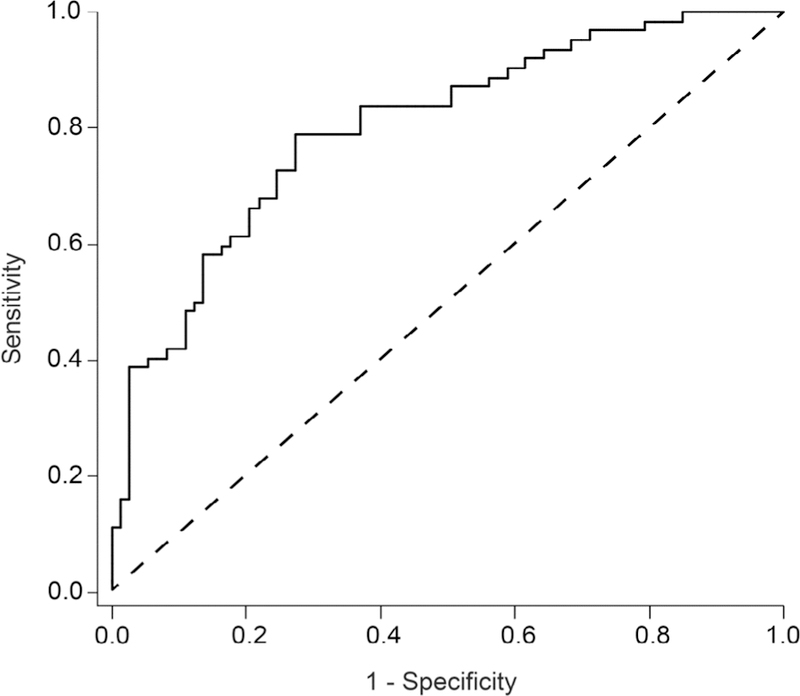
Receiver operating characteristic (ROC) curve using logistic regression models to predict PD. This model incorporates sex, temporal outer, and the center 1mm ETDRS region to predict PD. The cross-validated C-statistic is 0.799 (see methods), with a 95% confidence interval of 0.7928 to 0.8046. We additionally calculated the odds ratio estimates for these three effects. Sex, temporal outer, and the center 1mm ETDRS region had point estimates (and 95% Wald confidence limits) of 0.094 (0.036 and 0.248), 0.948 (0.907 and 0.990), and 0.963 (0.937 and 0.990), respectively. All p-values were less than 0.05 for these effects.

Due to previous studies showing that females have thinner retinas, sex was added as a predictor to this model [35]. Supplementary Table 2 shows the single predictors of each effect. Following sex the center 1 mm and temporal outer ETDRS regions, were significant incorporation into this model, this revealed that sex, along with the center and temporal outer regions were significant in predicting PD (ROC curve shown in Figure 3), with the C-statistic at 0.800. Two-way interactions between sex, temporal outer, and the center 1mm were investigated, but none remained significant. Therefore, when scaled axial length measurements for temporal outer and center ETDRS regions are used, along with a sex correction, the predictive properties are substantially better as compared to the superior outer alone (C-statistic is 0.800 vs. 0.662).

## Discussion

In this work, we assessed interocular symmetry, foveal pit morphology, and axial length corrected ETRDS thickness maps in a model to help predict PD using foveal morphology. Our findings add to the growing literature on the topic of utilizing the retina as a non-invasive biomarker to study PD. While there have been reports of interocular asymmetry in foveal thickness in PD [12,20], we did not observe any asymmetry in our PD patients. Furthermore, while foveal morphology has been suggested to be altered in PD [12, 14], we also found no difference in foveal morphology in our PD cohort. These inconsistencies could be due to differences in measurement technique and indicate that a standardized approach would be beneficial in future work. This is particularly important for measurements of the foveal pit, for which there are numerous approaches in the literature [12,35–36,39–43].

If the retina is to be used as a biomarker for PD, a model needs to be established to provide the highest possible sensitivity and specificity to ensure reliability in a clinical setting. While the dopaminergic cells in the retina and the relationship to PD need to be further explored, the accessibility of the retina and relative ease of OCT scanning offers a great opportunity for possible early detection of this neurologic condition. In developing an ideal model for predicting PD, axial-length scaling and sex should be incorporated into this model. Scaling for axial-length would provide greater stringency to make sure the retinal measurements are as accurate as possible before providing a prediction of PD. This could significantly reduce the number of false positives or negatives for prospective PD patients with longer or shorter eyes. In addition, factoring for sex into the model provides further accuracy of the results. Our cross-validated statistics revealed that incorporating sex into our model changed the accuracy of PD prediction from 0.662 to 0.800 while also altering which specific ETDRS regions which were significant predictors of PD (superior outer to center 1mm and temporal outer). Larger and longitudinal studies need to be performed to accurately determine if the center 1mm and temporal outer region are predictors based on this representative population, or if these specific regions could be unique to predicting PD.

Our study had a number of limitations. First, we utilized a relatively small group of subjects. In addition, our cohort of PD patients did not express any visual symptoms that may be described in PD, and they were at varied stages of PD diagnosis. Perhaps in a more severely affected group of patients we may observe some of the retinal changes reported by other groups [15,18]. Also, while our subjects were age-matched, we did not have sex-matched controls, with most of the HC subjects being women. However, this latter point is unlikely to have affected the main conclusion as sex differences were incorporated into our predictive model.

## Conclusion

Our study demonstrates the need for additional analyses to be performed regarding retinal structure in PD using quantitative OCT analysis. We believe that the discrepancy between previously published data is due in part to the use of incorrectly scaled OCT data, and propose that OCT devices enable either an export of raw thickness data for offline manipulation or an onboard correction of individual differences in ocular biometry. In addition, uniformity in measurement technique is critical for data sharing and easier comparison of results across studies. Discrepancies and challenges aside, the role of the retina as a biomarker in PD remains an exciting space for continued research.

## Supplementary Material

Sup table

## Figures and Tables

**Table 1:** PD Demographics

Subject	Age* (years)	Sex	UPDRS Motor Examination^♯^	Age at PD Diagnosis	On Levodopa	Age when DOPA started	Other Medications
**JC_0702**	44	F	ND	40	Yes	43	
**JC_0703**^‡^	66	F	33	65**	Yes	65	Amantadine
**JC_0705**	65	F	10	60	No	--	Amantadine
**JC_0706**^‡^	57	M	ND	57	Yes	57	Ropinirole
**JC_0708**	53	M	13	51	Yes	51	
**JC_0709**^†^	51	M	16	43	Yes	46	
**JC_0710**	56	F	37	53	No	--	Ropinirole
**JC_0712**	54	M	8	53**	No	--	Amantadine; Pramipexole
**JC_0715**	87	F	35	84**	Yes	84	
**JC_0719**	77	M	24	75	Yes	76	
**JC_0721**	71	F	4	67**	Yes	67	
**JC_0724**	49	M	25	40	Yes	40	Ropinirole
**JC_0725**^†^	78	M	ND	73	Yes	75	
**JC_0727**	61	M	7	57	Yes	61	Ropinirole
**JC_0728**	68	M	16	57	Yes	62	
**JC_0729**	70	F	21	64	Yes	64	
**JC_0731**	76	F	11	74	Yes	74	
**JC_0733**^§^	79	F	ND	67**	Yes	67	Amantadine; Ropinirole
**JC_0737**	59	M	23	56	Yes	56	Entacapone; Ropinirole
**JC_0738**	71	M	16	69	Yes	69	
**JC_0739**	64	M	9	62	Yes	62	Amantadine; Selegiline
**JC_0743**	61	F	14	60	Yes	60	
**JC_0745**	64	M	ND	58	Yes	60	Trihexyphendiyl; Ropinirole
**JC_0748**	76	M	9	75	Yes	75	
**JC_0750**	56	M	13	53	No	--	Pramipexole
**JC_0950**	53	M	6	46	Yes	46	Amantadine; Rasagiline
**JC_0951**	70	F	19	69	Yes	69	
**JC_0953**	53	F	5	48	No	--	Amantadine; Rasagiline; Ropinirole
**JC_0955**	76	M	31	72	Yes	74	
**JC_0957**	58	M	14	57	No	--	Amantadine; Pramipexole
**JC_0958**	70	M	19	66	Yes	66	Amantadine
**JC_0959**	70	M	23	58	Yes	60	Amantadine; Ropinirole
**JC_0960**	69	F	16	68	No	--	Amantadine

* Age at date of imaging

** Official age of PD diagnosis unknown; recorded first visit to Froedtert & the Medical College of Wisconsin

♯ Unified Parkinson’s disease Rating Scale (UPDRS) motor examination score

§ = Glaucoma Suspect

† = Received Deep Brain Stimulation

‡ = Likely PD complicated with dementia; ND = No Data).

**Table 2:** Foveal metrics *Values are reported as mean ± 1SD. Measurements were compared using a two-tailed t-test with 71 degrees of freedom for the right eye, and 60 degrees of freedom for the left eye).

Foveal Metric	HC subjects	PD patients	p-value
Depth – OD (mm)	0.1078 ± 0.024	0.1083 ± 0.021	0.9208
Depth – OS (mm)	0.1087 ± 0.026	0.1079 ± 0.020	0.8968
Diameter – OD (mm)	1.817 ± 0.215	1.849 ± 0.211	0.5150
Diameter – OS (mm)	1.845 ± 0.206	1.835 ± 0.187	0.8321
Slope – OD (deg)	11.90 ± 2.79	11.67 ± 2.48	0.7138
Slope – OS (deg)	11.86 ± 3.04	11.70 ± 2.04	0.8055
Volume – OD (mm^3^)	0.0748 ± 0.027	0.0766 ± 0.026	0.7697
Volume – OS (mm^3^)	0.0781 ± 0.027	0.0752 ± 0.025	0.6673
