# Supplementary material for: Assessing Retinal Structure in Patients with Parkinson’s Disease
Source: J Neurol Neurophysiol. Author manuscript; Available in PMC 2019 May 1. (PMC6494090; doi:10.4172/2155-9562.1000485)
Supplement: Sup table [file NIHMS1017407-supplement-Sup_table.pdf]

			**Pearson Correlation Coefficients, N = 135**						
			**Prob > |r| under H0: Rho=0**						
	**Temporal**	**Superior**	**Nasal**	**Inferior**	**Temporal**	**Superior**	**Nasal**	**Inferior**	**Center**
Temporal	Inner	Inner	Inner	Inner	Outer	Outer	Outer	Outer	1mm
	1	0.92035	0.87872	0.9307	0.79504	0.6675	0.62501	0.6468	0.53239
Inner		<.0001	<.0001	<.0001	<.0001	<.0001	<.0001	<.0001	<.0001
Superior	0.92035	1	0.91963	0.9123	0.80917	0.79302	0.77805	0.68604	0.44184
Inner	<.0001		<.0001	<.0001	<.0001	<.0001	<.0001	<.0001	<.0001
Nasal	0.87872	0.91963	1	0.91714	0.71772	0.69383	0.77913	0.66135	0.53658
Inner	<.0001	<.0001		<.0001	<.0001	<.0001	<.0001	<.0001	<.0001
Inferior	0.9307	0.9123	0.91714	1	0.79616	0.70455	0.7449	0.7159	0.45063
Inner	<.0001	<.0001	<.0001		<.0001	<.0001	<.0001	<.0001	<.0001
Temporal	0.79504	0.80917	0.71772	0.79616	1	0.83556	0.74068	0.83197	0.38868
Outer	<.0001	<.0001	<.0001	<.0001		<.0001	<.0001	<.0001	<.0001
Superior	0.6675	0.79302	0.69383	0.70455	0.83556	1	0.8334	0.81621	0.28775
Outer	<.0001	<.0001	<.0001	<.0001	<.0001		<.0001	<.0001	0.0007
Nasal	0.62501	0.77805	0.77913	0.7449	0.74068	0.8334	1	0.82856	0.20071
Outer	<.0001	<.0001	<.0001	<.0001	<.0001	<.0001		<.0001	0.0196
Inferior	0.6468	0.68604	0.66135	0.7159	0.83197	0.81621	0.82856	1	0.30487
Outer	<.0001	<.0001	<.0001	<.0001	<.0001	<.0001	<.0001		0.0003
Center	0.53239	0.44184	0.53658	0.45063	0.38868	0.28775	0.20071	0.30487	1
1mm	<.0001	<.0001	<.0001	<.0001	<.0001	0.0007	0.0196	0.0003	

Supplementary Table 1: ETDRS Correlative Values (The correlation values of all ETDRS regions between all eyes imaged in this study).

	**Wald Lower 95% Confidence Limit for Adjusted**		**Wald Upper 95% Confidence Limit for Adjusted**	
**Effect**	**Odds Ratio**	**Odds Ratio**	**Odds Ratio**	**p-Value**
Temporal_Inner	0.975	0.951	1	0.0487
Superior_Inner	0.968	0.944	0.993	0.011
Nasal_Inner	0.977	0.955	1	0.0466
Inferior_Inner	0.972	0.948	0.997	0.03
Temporal_Outer	0.946	0.912	0.981	0.0028
Superior_Outer	0.949	0.919	0.979	0.0012
Nasal_Outer	0.961	0.936	0.988	0.0042
Inferior_Outer	0.957	0.926	0.988	0.0067
Center_1mm	0.979	0.96	0.999	0.0373
Sex F vs. M	0.217	0.107	0.439	<.0001

Supplementary Table 2: Single-predictor odds ratios (Single-predictor odds ratios for each effect of the ETDRS grid and gender for predicting PD. Foveal pit morphology metrics of depth, diameter, and slope were not predictive of PD, with p-values > 0.60).
